# Clinical characteristics of patients infected with SARS-CoV-2 in North Wollo Zone, North-East Ethiopia

**DOI:** 10.11604/pamj.2021.38.217.27859

**Published:** 2021-02-25

**Authors:** Alene Geteneh, Birhan Alemnew, Selamyhun Tadesse, Abebe Girma

**Affiliations:** 1 Department of Medical Laboratory Science, College of Health Sciences, Woldia University, Woldia, Ethiopia,; 2 Department of Biotechnology, College of Natural Sciences, Woldia University, Woldia, Ethiopia

**Keywords:** Clinical characteristics, SARS-CoV-2, COVID-19, Ethiopia

## Abstract

**Introduction:**

severe acute respiratory syndrome coronavirus 2 (SARS-CoV-2); the causative agent of coronavirus disease 2019 (COVID-19), pandemics has remained to be a public health emergency of international concern. This ongoing pandemic has resulted in the death of millions of people globally. About one and a half thousand people have died due to this illness in Ethiopia. The clinical presentations of the disease vary with geography and populations. We therefore aimed at investigating the clinical characteristics of patients infected with SARS-CoV-2 in North-East Ethiopia.

**Methods:**

real time polymerase chain reaction (PCR) was conducted for 18,112 individuals suspected of SARS-CoV-2 infection during June 27 to October 20, 2020, at Woldia university COVID-19 testing center. Recorded data of 372 Ethiopians who tested positive for SARS-CoV-2 infection at Woldia university COVID-19 testing center were retrospectively extracted and analyzed using SPSS v25.0. A P-value of < 0.05 was considered statistically significant.

**Results:**

nearly 2.1% of the screened participants were found positive for SARS-CoV-2 infection. Among them, three fourth of SARS-CoV-2 infected patients were male, with an overall median age of 30 years. About 85% of the patients were asymptomatic. The most common clinical manifestations were cough (14.2%), followed by fever (11.0%) and headache (8.6%), whereas hypertension (1.6%), human immunodeficiency virus (HIV) (1.6%) diabetes mellitus (1.1%), and chronic respiratory diseases (1.1%) were relatively the most common comorbidities noted. The case-fatality ratio was found at 1.6%. Age and underlying comorbidities had a statistically significant association with severity and poor outcome of patients (P < 0.001).

**Conclusion:**

the finding from this study indicated that older age and people with underlying comorbidities are at high risk of having the severe disease and poor outcomes. Hence, appropriate care and priority should be given to these people to decrease the morbidity and mortality caused by this illness. The presence of higher asymptomatic infection is the possible indicator of potential asymptomatic transmissions within the community. This highlights the need for widespread testing, and contact tracing to flatten the transmission curve.

## Introduction

The ongoing pandemics of coronavirus disease 2019 (COVID-19), which is caused by severe acute respiratory syndrome (SARS-CoV-2) has remained to be a global challenge [1] with a progressive vaccine trial. Since December 2019, this horrific incidence continues to spread across the world like a wildfire. Nearly 48.5 million confirmed cases have been reported from 189 countries and more than 1.2 million deaths were documented so far (as of November 5^th^, 2020). Though the outbreak is sustained globally, there are expectations of second and possibly third SARS-CoV-2 waves of infections [2,3]. Some countries that suppressed the initial outbreaks are now already seeing infection rises again; “second wave”, which makes the illness to be still considered as “public health emergency of international concern” [4,5].

Relative to other regions, Africa had few numbers of individuals infected with SARS-CoV-2 [6]. The very young-aged population, harsh climate, impaired SARS-CoV-2 receptor (angiotensin-converting enzyme-2) gene expression, ethnic background, past infection with other coronaviruses, malaria, and other infectious diseases priming the immune system to fight new pathogens like SARS-CoV-2 are hypothesized to have a potential protective role for having relatively lower COVID-19 infections in the region [6-9]. Despite these, the poor socio-economic and poor health care system along with the absence of advanced laboratory settings may worsen the COVID-19 future outbreak in Africa. As of November 05^th^, 2020, nearly 98 thousand, and more than one thousand five hundred people have infected and died due to COVID-19 respectively in Ethiopia [10].

In most cases COVID-19 is transmissible at the early stages of the illness, screening people with classical symptoms is an ultimate determinant of whom would be quarantined and be tested [11,12]. Since SARS-CoV-2 infection symptoms vary from population to population [13], it is very important to have a set of different symptomatic, demographic, exposure, and behavior related data for early screening and fighting of COVID-19 pandemics. The key clinical presentations of patients with COVID-19 were fever, cough, fatigue [12,14], dyspnea, and sputum production [14]. These clinical features may or may not be consistent with SARS-CoV-2 infected populations in Ethiopia as the clinical features of COVID-19 vary between populations. Despite this variability, no study has described the clinical characteristics of COVID-19 patients in Ethiopia. Hence, the current study aimed to investigate the clinical characteristics of COVID-19 infected patients in North-East, Ethiopia.

## Methods

**Patients’ eligibility and data collection:** a total of 18,122 suspected individual were screened for SARS-CoV-2 infection during the four months study period. A pharyngeal swab was collected and used for SARS-CoV-2 detection as part of community surveillance from June 27^th^ - October 20^th^, 2020, at Woldia university COVID-19 testing center. All laboratory-confirmed SARS-CoV-2 infected Ethiopians with registered clinical signs and symptoms were retrospectively included in this study. A total of 372 patients were screened and included in this study. Demographic characteristics like age, sex, travel history, etc., clinical characteristics like symptoms and comorbidities etc of each patient were obtained from COVID-19 test request and reports record.

**Real-time PCR (RT-PCR):** the viral nucleic acid was extracted from the patient pharyngeal swab specimens using a viral RNA extraction kit (BGI, China) according to the manufactures´ instruction. The primer and probe used were specific for ORF1ab and N of the SARS-CoV-2 genome. The ORF1ab targeting primer were 5'CCCTGTGGGTTTTACACTTAA3' (F), 5'ACGATTGTGCATCAGCTGA3'(R), and the fluorescent probe (P) was 5'-FAM CCGTCTGCGGTATGTGGAAAGGTTATGG-BHQ1-3'. Target 2 (N), forward: 5'-GGGGAACTTCTCCTGCTAGAAT-3', reverse: 5' CAGACATTTTGCTCTCAAGCTG-3', and the probe was 5'-FAM TTGCTGCTGCTTGACAGATT-TAMRA-3'. The final reaction mixture was 30 μL comprising of 20 μL PCR mix (18.5 µL SARS-CoV-2 and 1.5 μL enzyme mix) and 10 μL template RNA. The thermal cycling was 50°C for 20 minutes, 95°C for 10 minutes, and 40 cycles of 95°C for 15 seconds and 60°C for 30 seconds [15,16].

**Data analysis:** data were checked for completeness and entered into Microsoft Excel, and transferred into SPSS v 25.0 for analysis. Variables of interest were summarized as frequencies and percentages. Bivariate logistic regression was used to test for association between different variables. Patients’ outcome and disease severity were used as dependent variables, whereas demographics and clinical characteristics were used as independent variables. Tests with a p-value of < 0.05 were considered statistically significant.

**Quality control:** for each laboratory work both positive and negative controls were run in parallel to make sure the test was free from false positive and false-negative results. The known viral suspension and molecular grade water were used as positive and negative controls respectively. Before and after each master mix preparations, the biosafety cabinet was cleaned with 10% bleach and with 70% alcohol, and finally radiated with ultraviolet for 30 minutes to prevent any contamination. To prevent any cross-contamination, laboratory works such RNA extraction, master mix preparation, template addition, and sample storage was conducted in separated rooms. The RT-PCR protocol was validated by the national reference laboratory, Ethiopian public health institute (EPHI) before the national surveillance program.

**Ethical approval:** before reviewing the laboratory request and report records, waiver consent was obtained from Woldia University. Woldia University Institutional Review Board has approved the testing protocol with protocol number WDUIRB03/20. A formal letter of permission and support was also obtained from Woldia University and Amhara Public Health Institute. The consent to participate in the study were not applicable as the study is retrospective. The CDC China was used as a reference guide of the testing protocol.

## Results

**Demographic profiles:** a total of 372 laboratory-confirmed patients were enrolled in this study. Among the patients, almost all of them had non-severe SARS-CoV-2 infections except 13 patients who were admitted to the hospital for severe illness. Three-quarters of the patients were males (n=279) and the median age of patients was 30 years varying between 5years and 85 years (Table 1). Nearly, 91% of the patients were aged <50 years old. More than 95% (n=354) of the cases were screened as part of the community surveillance, while 4.3% (n=16) of the cases were COVID-19 suspects. Two of the cases were tested due to their contact history. The underlying comorbidities were presented in 5.1% (n=19) of the SARS-CoV-2 infected patients. Hypertension (n=6), HIV (n=6), diabetes mellitus (n=4), chronic respiratory diseases (CRD) (n=4) and chronic cardiac diseases (CCD) (n=2) were the underlying diseases noted in these patients (Table 1).

**Table 1:** bivariate analysis of demographics and comorbidities with the disease severity, October 2020, North-East Ethiopia

		Severity of disease				p-value
		Asymptomatic	Mild	Moderate	Severe	
Age, median(range)	30yrs (5-85)	315(84.7)	41(11)	3(0.8)	13(3.5)	0.004
Age groups-No. (%)	<30yrs	181(57.5)	19(46.3)	1(33.3)	2(15.4)	<0.001
	30-49yrs	106(33.7)	21(51.2)	2(66.6)	5(38.5)	
	50+yrs	35(11.1)	1(2.4)	0	6(46.1)	
Sex	Female	82(26)	7(17.1)	1(33.3)	3(23.1)	
	Male	233 (74)	34(82.9)	2(66.7)	10(76.9)	0.639
Comorbidity	19(5.1)	9(2.9)	3(7.3)	0	7(53.9)	<0.001
Hypertension	6(1.6)	2(0.6)	1(2.4)	0	3(23.1)	<0.001
Human immunodeficiency virus	6(1.6)	3(0.95)	2(4.9)	0	1(7.7)	<0.001
Diabetes mellitus	4(1.1)	2(0.6)	0	0	2(15.4)	0.082
Chronic respiratory diseases	4(1.1)	2(0.6)	0	0	2(15.4)	<0.001
Chronic cardiac diseases	2(0.5)	0	0	0	2(15.4)	<0.001

**Clinical characteristics:** of the confirmed SARS-CoV-2 infected patients, fifty-seven (15.3%) of them had experienced at least one sign and symptom. Relatively, the most frequent presenting symptom was cough (14.2%), followed by fever (11.0%) and headache (8.6%). On other hand, hypertension (1.6%), HIV (1.6%), diabetes (1.1%), and chronic respiratory diseases (1.1%) were the major medical comorbidities observed in this study. The role of underlying diseases on the outcome of patients was assessed. Based on the preliminary bivariate logistic regression; the age of patients and the existence of comorbidities were statistically significant with the outcome of patients (Table 2, Table 3).

**Table 2:** bivariate analysis of demographic and clinical characteristics of COVID-19 patients with their outcomes, October 2020, North-East Ethiopia

Variables	group	Outcomes		P-value	OR	95%CI
		Death	Recovery			
Age	< 30 years	0	203	<0.001	0.97	0.867, 0.95
	30-49 years	2	132			
	50+ years	4	31			
Sex	Female	2	91	0.637	0.662	0.119,3.67
	Male	4	275			
Clinical sign and symptoms	Yes	6	51	0.993	190055863	0.0, -
	No	0	315			
Comorbidities	Yes	5	14	<0.001	125.7	13.75, 1148.8
	No	1	352			
Severity	Asymptomatic	0	315	0.988	0.0	0.0, -
	Mild	0	41			
	Moderate	0	3			
	Severe	6	7			

**Table 3:** cross tabulation of demographic and clinical presentations of COVID-19 patients with their outcome, October 2020, North-East Ethiopia

Variables			Outcomes	
			Death	Recovery
	<30 years		0	203
Age	30-49 years		2	132
	50+ years		4	31
Sex	Female		2	91
	Male		4	275
Sign and symptoms	Cough	Yes	6	47
		No	0	319
	Fever	Yes	5	36
		No	1	330
	Dyspnea	Yes	5	21
		No	1	345
	Sore throat	Yes	3	8
		No	3	358
	Headache	Yes	6	26
		No	0	340
	Easy fatigue	Yes	3	4
		No	3	362
Comorbidities	Hypertension	Yes	2	4
		No	4	362
	HIV	Yes	1	5
		No	5	361
	Diabetes mellitus	Yes	1	3
		No	5	363
	CRD	Yes	1	3
		No	5	363
	CCD	Yes	2	0
		No	4	366

## Discussion

The COVID-19 pandemic remains a global challenge affecting more than 48 million and has resulted in above 1.2 million deaths worldwide as of November 5^th^, 2020 [10]. SARS-CoV-2 infected patients manifest differently between populations, sex, race, age groups, ethnicity, and the presence of underlying diseases [17]. The most frequent presenting symptoms in this study were cough (14.2%), followed by fever (11.0%), and headache (8.6%) (Table 2). This was very lower compared to the meta-analysis of 4,203 patients with COVID-19 in China, where fever, cough, and dyspnea (80.5%, 58.3%, and 23.8%, respectively) were noted as the most frequently displayed clinical presentations [18]. The frequency of clinical manifestations of the current finding was very lower as compared to the single-center study in Wuhan, China; that documented fever (98.6%), fatigue (69.6%), and dry cough (59.4%) as the common symptoms of COVID-19 patients [19].

The frequency of clinical presentations varies with geography and study populations; in the study of 1,420 European patients with mild to moderate COVID-19 disease, the most common symptoms were headache (70.3%), loss of smell (70.2%), nasal obstruction (67.8%), cough (63.2%), myalgia (62.5%), rhinorrhea (60.1%), sore throat (52.9%), and fever (45.4%) [20]. This much discrepancy of clinical symptoms between studies [18,19] could be due to differences in study populations; race, ethnicity, sex, and other host factors [17]. The higher prevalence of asymptomatic (possibly presymptomatic) infections may be the reason for having a lower percentage of clinical manifestations in this study. Hypertension (1.6%), HIV (1.6%), diabetes (1.1%), and chronic respiratory diseases (1.1%) were the main comorbidities in this study. This was also lower to a result found in the meta-analysis in china; where hypertension, cardiovascular disease, and diabetes (16.4%, 12.1%, and 9.8% respectively) [18] were the most common comorbidities noted.

There are shreds of evidence of a higher magnitude of presymptomatic (asymptomatic at screening point and developed symptoms during follow-up) and asymptomatic infections. The magnitude of asymptomatic SARS-CoV-2 infections varies by up to 87.9% depending on the study sample size (higher with lower sample size) [21]. A recent systematic review and meta-analysis study has generated data of 48.9% and 15.6% pooled prevalence presymptomatic and asymptomatic infections [22] respectively. Another systematic review has also indicated 20% of SARS-CoV-2 infected patients remain asymptomatic throughout infection with a prediction interval of 3%-67% [23]. Similarly, a study in Japan showed a high (17.9%) proportions of asymptomatic infections [24].

Knowing the proportions of asymptomatic infections is very critical to estimate the true burden of the disease, and better understand and predict the transmission potential. In our study, nearly 85% of the patients were asymptomatic (could be presymptomatic). Our study has reported higher proportions of asymptomatic (possible presymptomatic) infections as compared to the above studies [22-24]. We can estimate that a large number of community members were at risk of getting infected with these asymptomatic patients in our context, as there is evidence of asymptomatic transmissions [21,23,25,26]. The case fatality ratio (CFR) in this study was 1.6% which is in agreement with June 30, 2020, national report and it was in the range for sub-Saharan African countries who reported death (CFR varied from 0.22% in Rwanda to 8.54% in Chad) [27]. To the best of our knowledge, this study is the first to describe the frequency and clinical presentations of asymptomatic SARS-CoV-2 infections in North-East Ethiopia. However, we were unable to trace laboratory findings that briefly show their hematological profiles, renal function test, and liver function enzymatic tests, which could be supportive in characterizing SARS-CoV-2 infections in our study settings.

## Conclusion

Of the patients with signs and symptoms, the relative frequency of cough, fever, and headache were 14.2%, 11.0%, and 8.6% respectively. Commodities such as hypertension (1.6%), HIV (1.6%), diabetes (1.1%), and chronic respiratory diseases (1.1%) have shown a correlation with the severity of disease with a statistically significant association with death. The majority of the SARS-CoV-2 infections were asymptomatic making the community more at risk of being infected. As per the findings from the current study, we can conclude that there is a high number of asymptomatic COVID-19 cases in Ethiopia and the effect of the illness is higher among persons with underlying co-morbidities. All in all, there is a need for scaling up of SARS-CoV-2 testing and contact tracing of asymptomatic cases that would have a role in finding the asymptomatic infection transmission hotspots and breaking the chain of transmission. This will probably decrease the transmission rate and the effect of the outbreak in Ethiopia. Further prospective studies with laboratory assessments should be conducted, and check the protective role enzymes and hormones on the severity and outcome of SARS-CoV-2 infected patients.

### What is known about this topic

- The most common manifested symptoms were cough, fever and headache though the proportion was too low;
- The common comorbidities in Ethiopia were hypertension, cardiovascular disease, and diabetes with a lower proportion as compared to studies in other countries;
- Older age and people with underlying comorbidities had a statistically significant association with severity and poor outcome of patients.

### What this study adds

- To the best of our knowledge, this is the first study to investigate clinical characteristics of patients infected with SARS-CoV-2 in North-East Ethiopia. Majority of patients (about 85%) infected with SARS-CoV-2 were asymptomatic;
- The other important issue addressed is the SARS-CoV-2 transmission, knowing the proportions of asymptomatic infections is critical to estimate the true burden of the disease, and better predict the transmission potential in Ethiopia;
- It is very critical to recommend for other researchers looking for the role of enzymes and hormones for being asymptomatic.
